# Preparation and Characterisation of Sustainable Wood Plastic Composites Extracted from Municipal Solid Waste

**DOI:** 10.3390/polym13213670

**Published:** 2021-10-25

**Authors:** Shahnaz Shahani, Zhongquan Gao, Mumtaz A. Qaisrani, Naveed Ahmed, Haseeb Yaqoob, Fuad Khoshnaw, Farooq Sher

**Affiliations:** 1 School of Energy and Power Engineering, Xi’an Jiaotong University, Xi’an 710049, China; shahnaz.shahani@outlook.com; 2 Department of Mechanical Engineering, Khwaja Fareed University of Engineering and Information Technology, Rahim Yar Khan 64200, Pakistan; mumtazqaisrani@yahoo.com (M.A.Q.); haseeb.yqoob@kfueit.edu.pk (H.Y.); 3 US Pakistan Centre for Advanced Studies in Energy (USPCAS-E), National University of Sciences and Technology, Islamabad 44000, Pakistan; naveed814@yahoo.com; 4 School of Engineering and Sustainable Development, Computing, Engineering and Media, De Montfort University, Leicester LE1 9BH, UK; Fuad.Hassankhoshnaw@dmu.ac.uk; 5 Department of Engineering, School of Science and Technology, Nottingham Trent University, Nottingham NG11 8NS, UK

**Keywords:** sustainable chemical engineering, wood plastic, composite materials, municipal solid waste, extrusion, sustainable polymers, moulding process

## Abstract

Municipal solid waste (MSW) contains plastic waste that can be used as a sustainable green substitute to reduce oil footprints, CO_2_ emissions, and environmental pollution. This study aims to recycle plastic waste by manufacturing wood-plastic composites and to improve its mechanical properties by using additives, coupling agents, and lubricants. These composites are prepared by mixing 40–70% of wood flour with 20–25% of a polymer matrix. Wood was degraded at 220 °C, and then the composites were processed at 50 °C. The manufacturing process carried out in the study involved wood waste meshing, drying, shredding, drying, trimming, filling, blending, compounding, and extrusion moulding. The compounding of composites was accomplished in twin-screw extruders. Once the mixture was uniformly mixed, its final shape was given by a two-step extrusion moulding. Previously, researchers aimed at enhancing the mechanical properties of the composites, but our research focus was to improve their durability for different industrial applications. The results suggest that the impact strength is 17 MPa with 50% of wood powder ratio while the maximum value for the tensile strength is 32.5 MPa. About 50% of an increase in wood powder resulted in 8.1% bending strength increase from 26.1 to 32.8 MPa. Reducing the plastic matrix and the wood-particles water swelling ratio resulted in better mechanical properties. The wood species also affected the mechanical properties with their excellent dimensional stability and less variability. A high proportion of wood fibre tends to increase its steady-state torque and viscosity. The mechanical properties against different wood-flour proportions indicate that composite materials exhibit superior water swelling behaviour and extrusion quality.

## 1. Introduction

At present, the world is facing environmental issues due to the municipal solid waste (MSW) generation by-products of human activities. As resource consumption has increased, the problem of high production and non-biodegradability of plastic is the reason behind the rapid reduction of natural resources. The World Bank’s static data show that 1.3 billion tons of MSW is produced worldwide and it is expected to increase to 2.2 billion tons by 2025 and could reach 3.40 billion tons by 2050 [1,2,3]. About 6.5 billion tons of plastic wastes are produced every year globally in the form of disposed polythene bags, masks, and water bottles [4,5]. As a result, all of these wastes have badly interrupted the food chain of marine life and terrestrial animals in nature [6,7]. At present, this problem of plastic pollution can be solved by the development of degradable plastic as a substitute for daily life use by both landfill and incineration processes [8,9,10]. However, these methods would cause environmental pollution with their waste residues [11,12]. On the other hand, a massive amount of wood waste generated in factories worldwide during manufacturing processes is commonly disposed to landfills; however, its decomposition process takes a significant amount of time. This is the reason today researchers are turning their interest to reuse wood waste and plastic waste (which makes up the largest proportion of solid waste globally from MSW) rather than burning or dumping.

However, developing this research brought the challenge of utilizing wood and plastic wastes as potential raw materials from MSW, significantly resulting in WPC’s production as an affordable product for environmental sustainability by considering its physical and mechanical properties [13,14]. WPC is advantageous for being glue-free and having zero carbon emissions during its manufacturing process [15]. In the early 1990s, many countries began to use waste plastics for WPC manufacturing [16]. In recent years, various studies reported this technique as innovative to study mechanical properties of WPCs prepared by different plastic wastes combined with different wood flour proportions of wood waste to formulate excellent properties and the best performance of WPC [17,18]. These materials are used for sustainable development to overcome MSW generation with the concept of compatibility, low thermal conductivity, non-toxic, low density, high durability and represents good mechanical properties [19,20]. Its recyclability and cost-effectiveness are gaining a greater source of attraction in the automobile and aerospace industries and for sports, home appliances and infrastructure applications [21].

WPCs are composed of a natural fibre/plant fibre and polymer matrix divided into thermoplastic and thermosetting polymers [22]. Plant fibre is the main focus of researchers due to its low cost, and biodegradable characteristics possess good mechanical and physical properties [23]. Researchers have investigated various compositions of wood and plastics. However, the current composition has not been investigated particularly in the context of mechanical properties. Therefore, mechanical properties of WPC such as bending strength, tensile strength, impact strength, and water absorption tests were investigated according to ASTM standards. The composites consist of 40–70% wood flour and 25–55% polymer matrix (polyvinyl chloride, polypropylene, polyethylene). These are manufactured from recycled materials which can be fixed and machined in the same manner as wood [24]. The results propose that WPC’s are advantageous in terms of high efficiency, better stability, and moisture resistance. An optimum amount 50% of wood flour is cooperated to plastic constituents to obtain interfacial compatibility which provides a maximum impact strength value of 17 MPa, tensile strength of 32.5 MPa, and bending strength which increased to 32.8 MPa.

The mechanical properties can be enhanced by appropriate size selection of raw materials and incorporation of additives, coupling agents, and pigments during the entire process. An extra possibility for decreasing the breakability is compounding WPC with strengthening agents. The organic silicon, phosphate, organic phosphorus-nitrogen flame retardants in polyethylene (PE) makes wood-plastic composite materials with good flame-retardant effects, toughness, chemical corrosion resistance, and easy moulding. Elevated temperature and moisture exposure are the major factors that affect the mechanical properties of WPC under varying responses to environmental conditions. Reducing the water swelling ratio between the plastic matrix and the wood-flour particles shown better mechanical properties. The prepared WPC’s surface and cross-section morphology was examined by scanning electron microscope (SEM) micrographs that shown adhesion interaction between composites considerably strengthen the WPC’s mechanical properties.

## 2. Composition of Wood Fibres and Polymer Matrix

WPC made from wood and plastic wastes will decrease the MSW generation, lower the plastic price, and enhance the properties of composites overall when the natural fibre is mixed with plastics. The utilization of these wastes instead of virgin ones is more meaningful from the environmental point of view.

### 2.1. Wood Fibre

**Figure 1:**
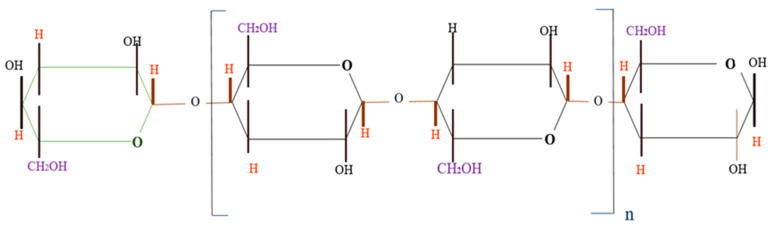
The chemical structure of cellulose.

The composition of wood flour is very complicated as it is a sensitive ingredient that affects the thermoplastic material and protects it from moisture. The lignin, cellulose, and hemicellulose content have strong influences on the mechanical properties of the materials. Wood fibre is advantageous in terms of ecological characteristics, degradability, low cost, and low energy consumption. The chemical structure is a linear polymer compound of cellulose, which is the main provider of the mechanical properties of plant fibre. All of the plant fibres have crystalline alike structures constituting about 65–70% cellulose [25]. The chemical structure of cellulose presented in Figure 1 is a natural polysaccharide whereas D-glucopyranose rings are connected with glycosidic linkages [26]. Moreover, the end characteristics and properties of cellulosic fibres are then altered by adding lignin and further non-cellulosic elements.

### 2.2. Polymer Matrix

The recycled fractioned plastic shown in Table 1 is classified according to melt flow indexes (MFI) high, medium or low obtained from MSW consists PP, PE, PVC, and polyolefin [27]. To obtain plastic grades of higher quality their treatment included size reduction, washing, elimination of metallic elements, and regrinding. They have better inter laminar strength, short processing time, absorb less moisture and resistance to impact. They have good chemical resistance, creep, and higher thermal stability [28].

### 2.3. Interfacial Bonding between WPC Composites

Different polarities make the composites incompatible, resulting in poor interfacial bonding between them. The coupling agent produces chemical bonds, polymer molecular entanglement, and mechanical locks. The poor incompatibility results in weak interfacial defects between thermoplastics and the wood fillers which leads to poor mechanical properties of composites [29,30]. The natural weathering conditions cause interfacial strength degradation influence, water absorption effects, and mechanical properties of WPCs reported in the literature [31,32]. The mechanical properties of composites during their manufacturing depend on the type of wood species, the compounding, moulding temperature, and pressure conditions [33]. Different researchers described the difference between WPC produced from different morphology, the chemical composition of fibre, arrangement of microstructure, packing, mechanical bonding between particles, interlocking, stress transfer efficiency of wood fibre matrix, and surface chemistry as well as surface energy [33]. The chemical constituents, mechanical and physical properties of the plant fibres or cellulosic fibres are shown in Table 2 [34].

## 3. Experimental and Machining Processes of WPC

**Figure 2:**
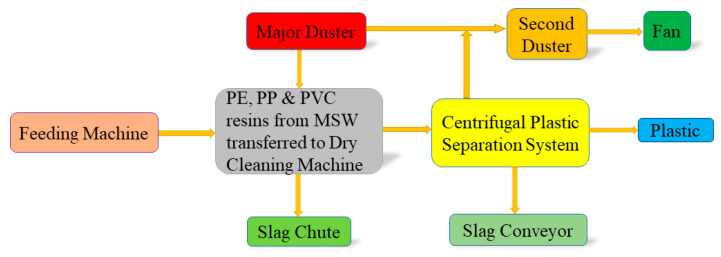
Preparation process flow chart of wood-plastic composites extracted from MSW.

The machining equipment consists of a process that was carried out by high-speed mixer device (Shr-50a): Zhangjiagang Acer Machinery Co.; CMT6104, screw granulator crusher, non-inter meshing and mixing was followed by a co-rotating twin-screw extruder supplied by: Zhangjiagang Acer Machinery Co. Ltd., China, in order to make compound into pellets. WPC machining process includes wood waste meshing, drying, extruding, shredding, drying, trimming, filling, blending, adjusting thickness injection moulding, and extrusion moulding processes. The natural fibres used were waste wood flour collected from Xian Hansen walled furniture market, China. Polymers used for the process were polyethylene PE, polypropylene PP, and polyvinyl chloride PVC obtained from Xi’an village of garbage plant which collects MSW. Waste plastic was cleaned by the process illustrated in Figure 2. The process starts with a feeding machine PE, PP, PVC resins from MSW transferred into the self-designed high alloy gold tooth type dry cleaning machine. Then it is transferred to the plastic separation system through the spiral gear high-speed collision light plastic alloy. The impurities are separated from the slag chute. Then, it is transferred to a centrifugal separation system to get clean plastic, and then into the aggregate preparation of plastic particles.

**Figure 3:**
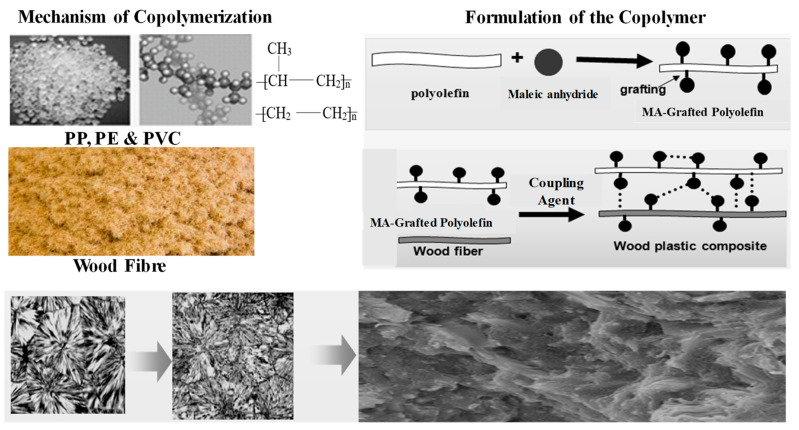
Multi copolymer technology; analysis and formulation mechanism of the copolymerization.

Figure 3 shows WPC manufactured from wood waste and plastic waste 40–70% wood particles, at density 0.386 g/cm^3^ crushed into 20, 40, 60, and 80 meshes mixed with 25–55% polymer matrix (PP/PE, and PVC). PVC is a white or light-yellow hard powder, with a density of about 1.40 in the range of 80–85 g/cm^3^ used. The density of PP/PE is between 0.9–0.91, in the range of 164–170 g/cm^3^ used. The coupling agent 3% by weight SILANE KH-550, obtained from Nanjing, was used to enhance the compatibility of the wood particles and the polymer matrix to fabricate WPC. Moreover, to introduce the polar maleic anhydride groups in polyolefin, the polyolefin with 3 wt.% maleic anhydrides grafted were mixed through a chemical bond then fixed to enhance the interface between the filler and polymer matrix. This could increase the flexural modulus and elastic modulus of the composite, and obviously improve the strength, stiffness, impact toughness, shape and size of the WPC. The coupling agent acts as a bridge that reacts with hydroxyls on the wood fibre and the grafted polyolefin. The polar wood flour and non-polar plastic combined well show a better interface. The production process of WPC was identified as a minimum pollution emitting process in terms of environmental standards.

### 3.1. Compounding and Synthesis of WPC Sample Extraction

**Figure 4:**
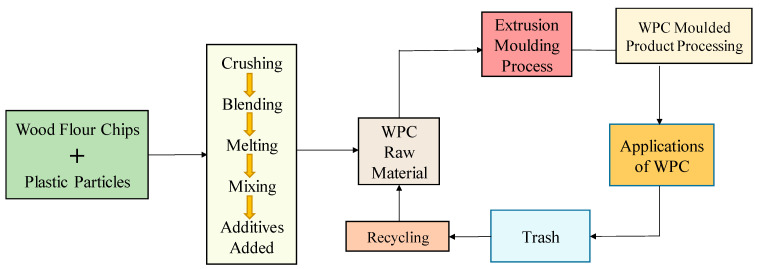
Compounding and Synthesizing of WPC with Extrusion Moulding.

WPC preparation was carried out by extrusion moulding process since injection moulding is an expensive process. Compounding and synthesizing of WPC with extrusion moulding are shown in Figure 4. In step one of the compounding processes, the wood flour is mixed with melted plastics to produce the wood-plastic materials; in step 2, the extrusion moulding process is heated and compressed into desired shapes.

**Figure 5:**
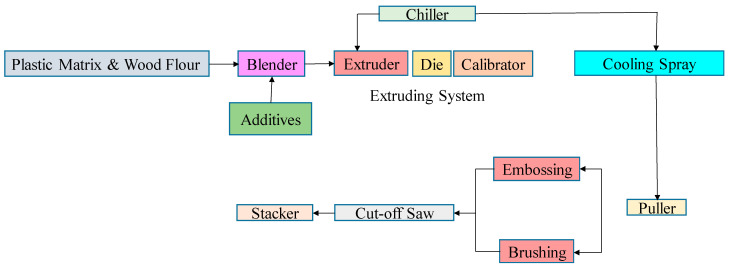
Process flowchart of WPC manufacturing process with extrusion system.

The flow chart of the WPC manufacturing process is demonstrated in Figure 5 with the extrusion forming process. The collected wood-flour sample was initially dried in an oven at 60 °C, moisturized, blended, weighed, degraded at 220 °C, and meshed in the form of sawdust particles. The adverse effects of the mixture were reduced then fed into the furnace at 140 °C for 4 h duration to avoid burning and to dry. After drying, it was mixed with talc and plastic in a container. The wood-thermoplastic mixture in pellet form was conveyed into a hopper that was fed to the extruder at 200 °C as a higher temperature may destroy its physical properties. Consequently, as the material entered the initial extruder zone, the screws warmed up and melted. The softened thermoplastic resin material was enforced out of a die to get its desired shape. The highly viscous molten composites needed heavy-duty equipment to force out of the die. To strengthen the thermoplastic material, it was chilled with a water spraying when it exited from the extruder with the desired pattern and then cut to a final length. Finally, desired WPC was obtained after processing at 50 °C, and then to increase the pressure of the product twin feed screws extruders with conical shapes were used.

### 3.2. Improvements in Mechanical Properties of WPC

The mechanical properties of WPC depend on the interaction between wood and thermoplastic materials, incorporating a coupling agent as an additive to improve interaction. Materials used in the WPC study are according to PP/PE chemical structure with above 90% purity. The additives such as colourants, stabilizers, 1.2% reinforcing agents, acetone, and ethylene were used. The paraffin wax 0.1% was employed as the lubricant. About 70% of different wood flour (pine and bamboo) particles and 25–30% of the polymer matrix (PP/PE, PVC) were used as composite materials. Photochemical ultraviolet (UV) stabilizers were used to protect the colour and prevent WPC from the UV radiation of sunlight. Antibacterial zinc borate was used as a wood preservative to protect composites from mycological attack. Fire-retardant chemicals were used to decrease the burning tendency of WPC. Coupling agents 3% used SILANE KH-550 obtained from Nanjing coupling agent limited enhanced the interaction between composites. Talcum powder 2.5%, and calcium carbonate 1.5–3 wt.% used were obtained from Jiangxi’s source powder limited. Stearic acid and stearic acid lead lubricants were obtained from Gaomi City, Kim Bok-Cheung Additives Co. Additives 0.8% were used to improve the processability of molten WPC mixture for the extrusion process. Antioxidants charcoal used was obtained from Shaanxi Larry New Resource Technology Company Limited. WPC with desired colours was furnished with pigments.

## 4. Results and Discussion

### 4.1. Effect on WPC Mechanical Properties

The samples were prepared for testing their mechanical properties using the test method ASTM GB series as per the National Standard series. The CMT6104 Electromechanical Universal Testing Machine supplied from Shenzhen New three Cisco Measurement Technology Limited was used to test bending and tensile strength. Charpy impact testing machine used to test the impact strength supplied from XJJ-5, Chengde City Kim Kun detection apparatus. BT-2003 granularity distribution supplied by Dandung city of hundreds of Instruments Co. Furthermore, laser scanner, melt index tester, pendulum impact testing machine, aging box, and SSX550 shimadzi scanning electron microscopy was used to get the microscopic views of matrix and wood fibre. WPCs manufactured using wood fibre, thermoplastics, and a small quantity of additives enhance mechanical properties with higher strength decrease density as compared to solid wood offer better acoustic performance, lowers the production cost, higher water, and decay resistance [35].

The evaluation of WPC performance was carried out by tensile, bending, and impact strength tests. The parameters that influenced the mechanical properties during its manufacturing process were the type of wood species, moulding temperature, and pressure conditions. Results suggest that mechanical properties against different wood flour proportions indicate that WPC exhibits superior water absorption behaviour and extrusion quality. Overall, moderate quantities of each composite material demonstrate higher mechanical properties for the good quality WPC is 50%. The result shows that the strength of WPC depends upon fibre matrix interaction and wood fibre surface chemistry that takes part in its strength improvement. The length of fibre particles and size makes an impact on mechanical properties. It has an excellent composition for humidity exposure. By incorporating the maleic anhydride grafted polypropylene (MAPP) the water uptake and thickness swelling can be significantly reduced [36]. The significance of coupling agent illustrated in many studies improves the mechanical properties as well as stability of the shape and size of the WPC [37]. Using a low-molecular-weight coupling agent tensile strength can be increased by better wetting the wood fibres. However, it caused minor declination of the flexural strength [38].

### 4.2. Effect on Bending/Flexural and Tensile Strength

**Figure 6:**
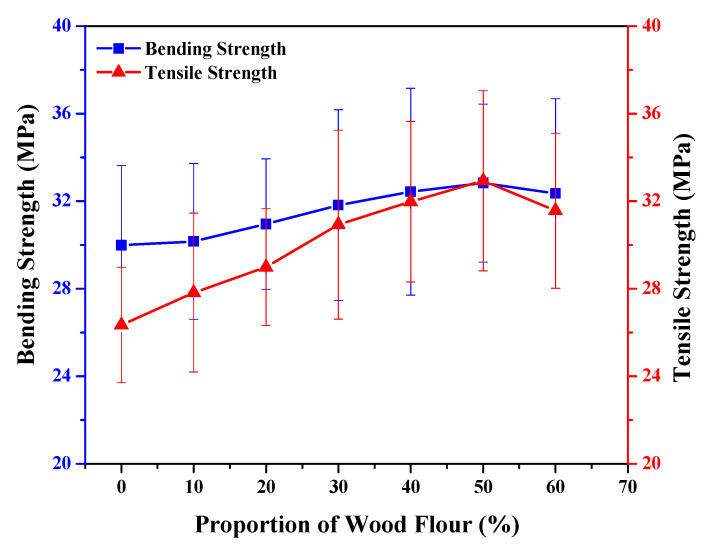
Bending and tensile strength at different wood flour proportions.

An electronic universal testing machine used was set at the speed of 10.87 mm/min. The three sample sizes about 500 mm of each length from different sections of the prepared WPC were taken for testing. To run the testing machine for data recording its span was adjusted to 420 mm, to get the average data test was repeated three times. Equation (1) is used to calculate bending strength or modulus of rupture (MOR). *MOR* increment rate calculated using Equation (2).

(1) $$MOR=\frac{3PL}{2bh^{2}}$$

(2) $$MOR\;Increment\;Rate=\frac{\left(MOR2-MOR1\right)}{MOR1}\times 100$$

where *P* is maximum loading (N), *L* is for the span which measures in mm, b is for the width of the test specimen which also measures in mm, and *h* is for the height of the test specimen measures again in mm. Figure 6 shows that the optimum amount of wood flour for the WPC is considered as 50% that shows strong interfacial interactions and strong adhesion between particles with efficient stress transferring capacity from matrix to wood fibre. The bending strength of the material increased around 25% with an increase from 26.1 MPa to a maximum value of 32.8 MPa as a result of enhancing the adhesion among components. When the wood powder percentage increased to 50% resulted in an increase in bending strength by 8.1% and a slight declination in tensile strength causing weak interfacial interactions. The reason is that with a relatively high torsional rigidity and strength of the sawing foaming powder surrounding the plastic substrate causes bound layer effect, the lock level to increase wood-plastic material rigid role, which is equivalent to the fibre material performance. The interfacial layer makes matrix and filler bind together to pass through stress, therefore, it has a significant influence on WPC properties. The strength of WPC’s $A=\pi r^{2}$ depends on the fibre end and the fibre’s pull-out process during interface failure [39]. This phenomenon is more noticeable as the fracture surface area increases cracks travel around the wood particles, with increasing the size of the particle. As a result of this, less energy is needed to fracture a specimen containing a larger size of particles [40].

For tensile strength data, three samples size of 80 × 40 × 6 mm from different sections of the prepared WPC were taken for testing. To start the test program an electronic universal testing machine adjusted to the span at speed 10.87 mm/min. The tensile strength can be calculated using Equation (3).

(3) ƍ=P/A

where *P* is the tensile load measured in *N*, *A* is the cross-sectional area measured in mm^2^, and ƍ is tensile strength measured as the capability of a material to survive in longitudinal stress or pressure. It defines the maximum stress that the material can survive without breaking up. It is calculated as a unit of force per cross-sectional area. The capability to withstand breaking under tensile stress can be identified as a vital property in material science and structural applications. The tensile strength and the bending strength depicted in Figure 5 show the wood proportion increased from 0 to 50%, resulting in maximum tensile strengths of 32.5 MPa and 32.8 MPa for the bending strength. It was measured as per ASTM D638 standards to minimize the errors with equipment. WPC affected by the filler fraction and interfacial adhesion formation in the particles resulted in a decrease in tensile strength. Based on results in the present study, previous studies also demonstrated that the increase in bending and tensile strength of WPC is due to the use of coupling agent MAPP, which improves the bonding compatibility of interfacial adhesion between hydrophobic polymer and hydrophilic wood flour leads to better mechanical properties of composites [41,42,43].

The tensile strength of WPC is affected by different proportions of wood flour and it becomes optimum at 50% of wood-flour proportion. The fibre material in addition to high stiffness and strength surrounding the plastic matrix causes the boundary layer effects. Tensile strength is enhanced by increasing the rigidity of WPC. After that modified hydroxyl has made the esterification reaction, which increases the mechanical performance of the material. As the wooden proportionate continues to increase, tensile strength begins to decrease due to the large wood fibre volume and the contact region between wood fibre surfaces which have an insufficient plastic surface. It was noticed that wood fibre containing droplets caused weak compatibility in many plastic substrates when reunification and destruction of the plastic substrate continuity occurred, causing degradation in the mechanical performance of the composites. The results of mechanical properties investigated in this study are consistent with previous research that increasing wood particle size significantly improves the tensile and bending strength of WPC [44].

### 4.3. Effect on Impact Strength

**Figure 7:**
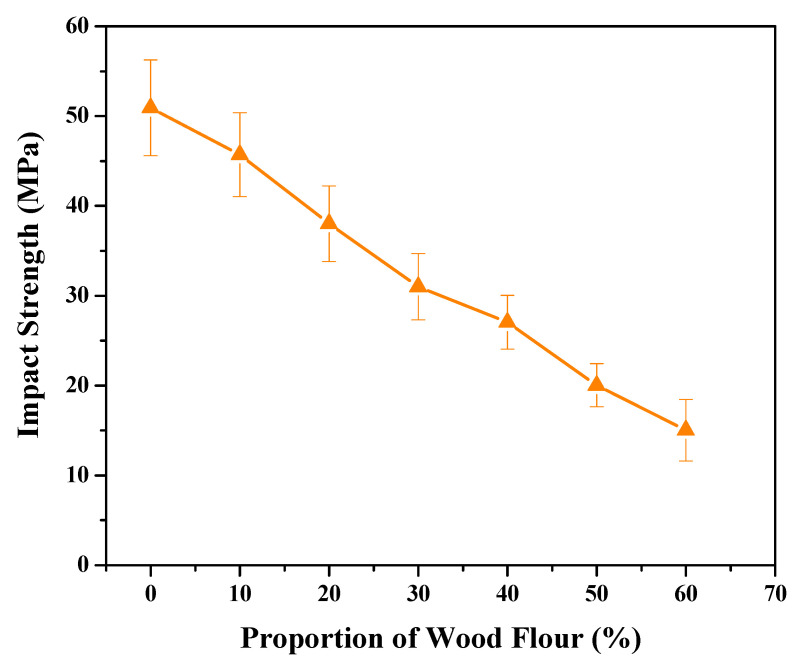
Impact strength via different wood flour proportions.

The pendulum impact testing machine switched on to the testing mode. Three samples size of about 80 × 10 × 4 mm from different sections of the prepared WPC were tested. The impact strength in Figure 7 drastically decreased, with the increment of wood flour moving from 0 to 60%. A higher proportion of wood flour tends to have lower impact resistance, due to the weak interfacial attraction between the wood particles and the plastic polymer. The addition of wood flour destroys the continuous dispersion of the plastic base which hampers the impact strength. The impact strength declined with composite deformation that affected the material fragility to change. Our results propose that the impact strength is more than 17 MPa with a wood powder ratio of 50%. The high-water absorption is considered a major drawback of natural fibre since it causes dimensional instability. Reduction in the water adsorption and swelling ratio indicates that the wood flour and the plastic matrix are interrelated to mechanical properties with a better physical and chemical attachment. Other researchers have shown that the addition of epoxy resin in the wood flour coupling agent mixture extruded the WPCs with a special screw arrangement which improved the mechanical properties of the composites. To reduce its water adsorption and improve its elastic modulus of composites there are several techniques proposed such as compression, extrusion, and injection moulding (from high to low) [45].

### 4.4. Micrograph Analysis of WPC Fracture Surface

**Figure 8:**
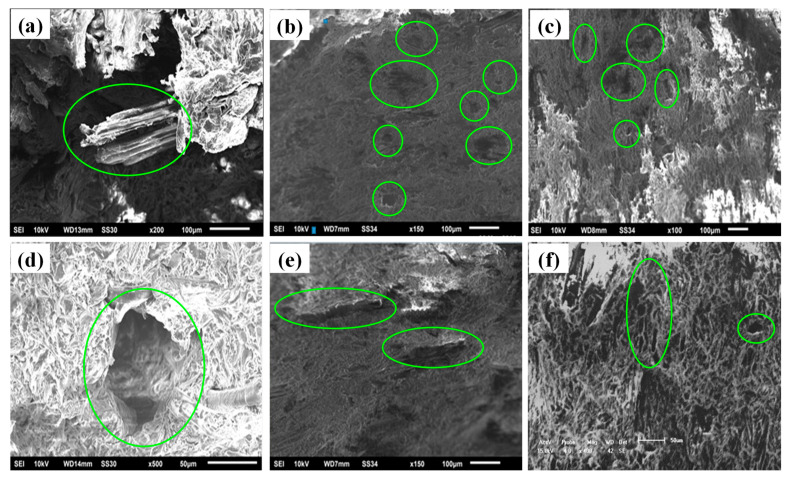
SEM interfacial adhesion between wood-flour and polymer matrix; (**a**) 40 mesh bamboo powder without coupling agent, (**b**) fracture surface of WPC made from 40 to 80 Bamboo Powder mesh, (**c**) effects by adding 0–3 wt.% calcium carbonate on mechanical properties of WPC, (**d**) wood powder penetrates the plastic matrix at 1 wt.% coupling agent, (**e**) effects of calcium carbonate on mechanical properties of WPC 1.5–2 wt.%, and (**f**) effects of calcium carbonate on mechanical properties of WPC 2.5–3 wt.%.

It is indispensable to observe the microscopic views in Figure 8; all SEM images show a better explanation of the fractured surface of WPC. In-depth microscopic analysis of SEM images provides interfacial adhesion between the different meshes of bamboo wood-flour and polymer matrix. Spacing between plastic polymer matrix and wood particles plays a pivotal role in its physical and mechanical properties. The large size of spaces available between the polymer and fibre could be observed from SEM images. The poor interfacial adhesion between the hydrophilic wood and the hydrophobic polymer causes ductility in composites. The development of biological activity in WPC penetrates its structure by accelerating the decomposition of the wood fibre. The formation of different size of voids exceeds wood fibre saturation under certain conditions of temperature. It becomes a storage location for free water after the moisture content initiates wood decay. The coupling agent calcium carbonate used can enhance the compatibility of the material interface between filler and polymer matrix which can increase the strength, stiffness, impact toughness, and stability of the shape and size of the WPC.

Figure 8a indicates that when the wood particles are of 40 mesh, without adding the coupling agent interaction between polar wood and non-polar wood particles, is very poor before its pre-treatment. It is difficult to form a good interfacial layer between the wood and plastic matrix when it is subjected to the bending strength to transmit a certain amount of force. The low mechanical properties of the material crack with a slight gap, causing a poor interface between the wood powder and the plastic matrix. Figure 8b represents that when the number of bamboo powder mesh increases from 40 mesh to 80 mesh, the static bending strength of the material increased from 26.1 MPa to a maximum value of 32.8 MPa. It indicates the interface between the wood powder and the plastic substrate is relatively good, so the mechanical properties of the material are relatively high. The wood powder left shows significant traces, indicating a poor interfacial layer that exists between the wood flour and the plastic matrix. When the material is smashed by the external force, the force is not transmitted well and the wood powder is pulled out of the plastic matrix. Therefore, the use of coupling agents enhances the mechanical properties for the good quality of WPC.

Figure 8c signify the effects by adding 3 wt% (3 kg) calcium carbonate. This slightly improves the thermal stability and mechanical properties of the WPC since by adding 0 wt% coupling agent, it causes small pores over the wood powder section that under the premise of wood powder plastic contents are in a material formulation. In Figure 8d wood powder penetrates the plastic matrix at 1 wt% coupling agent. The void left after the powder is then pulled out, indicating that there is no good interface between the wood powder and the plastic matrix at this time. Figure 8e shows the effects of adding coupling agent 1.5–2 wt.% adhered to the high-density polyethylene; the traces left by the wood powder are visible. Figure 8f shows the effects of adding calcium carbonate 2.5–3 wt.% in a large amount; it forms an agglomeration in the material, which destroys the interfacial interaction between the composites and forms many stress concentrations points (as shown in the images).

### 4.5. Water Swelling Ratio Analysis

This test was carried out as per ASTM D 570 to determine water absorption by weighing the samples at regular intervals. Each test sample’s weight was measured and then soaked in distilled water for 24 h. Then dry weight was taken again. The thickness of the sample was measured. Three samples of 100 × 100 mm from different sections of the prepared WPC were taken. The specimens were put in the (20 ± 2) °C, relative humidity of (65 ± 5)% condition to ensure constant mass and weighed. The specimens were immersed in a water tank which has 7 ± 1 pH at (20 ± 2) °C temperature. After 24 h ± 15 min, the specimens were removed from water to calculate the water absorption. Water absorption is calculated using Equation (4).

(4) WA=(Me−Mo)/Mo×100

where *Me,* represents the mass of the sample after immersion and Mo represents the mass of the sample before immersion. Swelling thickness can be evaluated using Equation (5).

**Figure 9:**
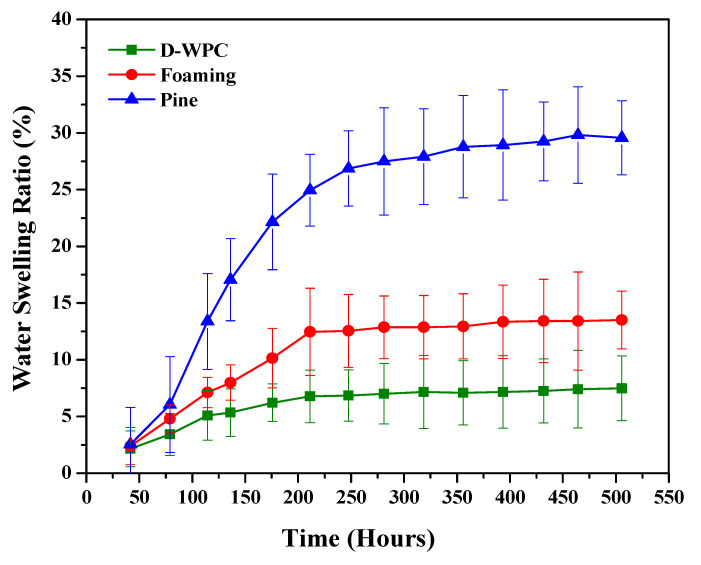
The effect on water swelling ratio with time for different types of wood fibre.

(5) TS=(te−to)/to×100

where *TS* represents thickness swelling, *te* shows the thickness of the sample after immersion soaking in water in mm and *to* is the thickness of the sample before immersion (measured in mm). If the wood fibre components cause higher water absorption, then it will be identified as a disadvantage of the natural fibre, weakening the bonds in the fibre. As a result, it will adversely affect the fibre-matrix interface. Further higher water absorption causes dimensional instability. Hence, researchers propose a coupling agent as a better solution for this problem in plant fibre. Figure 9 shows that the WPC water absorption is significantly low. The water swelling ratio against the number of hours for different types of wood fibre increases rapidly until 200 h.

Wood-flour has hydrophilic characteristics that can be reduced by covering the surface area with some paint. WPC produced by using pine tree flour as natural fibre has a higher water swelling ratio with better mechanical properties due to its inherent characteristics. If the water swelling ratio has a higher value, the material tends to bend and expand as a result of an increase in the intra-molecular spacing occurring in between WPC. This leads to having higher deformation and limits it in-between the applicability in various industries. WPC’s water absorption ratio indicates excellent results for agriculture, construction, and industrial applications. Extensive time is required to initiate fungal decay resistance resulting in wood weight loss. The storage of WPC in natural conditions is insufficient to promote fungal growth due to low water absorption. The water absorption of WPCs is correlated to wood fibre.

### 4.6. Comparison of WPC with Commonly Used Materials

A comparative analysis of WPC with steel, cement, and bamboo-like plant species is represented in Table 3. WPC has acquired numerous physical appearances that cause no pollution and can be recycled. Bamboo has higher water absorption, biodegradability, and light characteristics. However, the optimized unique two-layer structure of WPC strengthens it to last for more than ten years in terms of thermal insulation with better resistance to corrosion, microbial degradation, and UV decomposition. The cement can resist compressing but fails to resist bending and shearing strength. WPC is light and simple to install, as it does not cause any damage to the plastic film covered outside. WPC is a transitional renewable material for future demands. It can be used in various applications (e.g., outdoor decking and industries, including in electrical casings, packaging, for daily living supplies, and civil engineering) [46].

## 5. Conclusions

To overcome MSW generation, WPC (as the best alternative with better mechanical properties and hydrophobicity) was successfully prepared by extrusion moulding. The experimental analysis has shown that 50% of the wood-flour proportion resulted in a maximum increment of 32.5 MPa tensile strength, 32.8 MPa bending strength, and 17 MPa impact strength with dimensional stability when exposed to moisture. The particles’ cross-section design, wood types, temperature and pressure conditions affected the manufacturing process. The tensile strength affected interfacial bonding between composites, due to the presence of non-cellulosic compounds, and roughness at the surface of the wood fibre. The tensile strength of WPC intensely increased due to the better adhesion between the wood fibre and matrix using a coupling agent with low molecular weight. The water swelling ratio for different types of wood fibre increased rapidly until 200 h. Its higher value causes deformation as a result of an increase in the intra-molecular spacing which occurs in between WPC. The blend of hydrophilic wood filler without adding a coupling agent leads to poor interfacial bonding between them. The water absorption capacity is adversely affected by the nature of the wood filler when the thermoplastics entirely covers the wood filler, avoiding contact between them. The SEM analysis confirms that the strength and ductility of wood fibre reinforced composites affect the nature of the polymer matrix, the volume fraction of wood fibre, and the interfacial adhesion quality. We recommend comparing the mechanical properties of different types of plant species with different wood flour proportions. Chemical modifications and surface treatments are further needed to improve hydrophobicity and interfacial bonding in order to achieve better mechanical properties for WPC. The foaming technology, nanotechnology, and organic-inorganic hybrid all need to be further incorporated in order to enhance the cost-effectiveness of WPC.

## Figures and Tables

**Table 1:** The properties of virgin and fractionated reused polymers [27].

Polymers	Melting Temperature (°C)	Melt Flow Index at 230 °C (g/10 min)
Polypropylene (PP) Virgin, MH418	163	4.50
Polyethylene (PE) Virgin, I 668	134	5.5 (190 °C)
Polypropylene (PP)Homo-Polymer Medium MFI	164	7.30
PP Homo-Polymer High MFI	165	22.10
PP Filled Garden Fraction High MFI	162	11
Polyvinyl chloride (PVC) Polystyrene (PS) Polylactic acid (PLA)	163 240 150–160	7.27 2.0–16 4–8

**Table 2:** Chemical, physical, and mechanical properties of the plant-based or natural cellulosic fibres [34].

Fibres	Chemical Composition			Physical Properties			Mechanical Properties		
	Cellulose (wt.%)	Hemicellulose (wt.%)	Lignin (wt.%)	Moisture Content (wt.%)	Density (g/cm^3^)	Diameter (µm)	Tensile Strength (MPa)	Young’s Modulus (Gpa)	Elongation at Break (%)
Abaca	56–63	20–25	7–9	5–10	1.5	150–180	430–980	12	3–10
Bagasse	55.2	16.8	25.3	20–28	1.2	320–400	20–290	19.7–27.1	1.1
Bamboo	26–43	30	21–31	11–17	0.9	10–30	250–850	9.8	5.6–8.6
Banana	63–64	17–19	3–5	8–10	1.35	160–200	355	33.8	53
Coir	36–43	0.15–0.25	40–45	8	1.15–1.46	100–460	131–220	4–6	15–40
Cotton	82–90	5.7	-	7.85–8.5	1.5–1.6	12–38	287–800	5.5–12.5	7–8
Flax	71	18.6–20.6	2.2	8–12	1.5	40–600	88–1500	27.6	2.7–3.2
Hemp	70.4–74.4	17.9–22.4	3.7–5.7	6.2–12	1.47	25–500	550–900	70	1.6
Henequen	58–60	28–30	7–8	10–12	1.4	160–180	430–580	15–20	3–4.7
Jute	61–71.5	13.6–20.4	12–13	12.5–13.7	1.3–1.49	25–200	393–800	13–26.5	1.16–1.8
Kapok	35.5	22–45	21.5	9.86	0.29	30–36	50–90	2–5	1.8–4.3
Kenaf	35–57	21.5	15–19	6.2–12	1.2	30–50	295–930	53	1.6–6.9
Oil Palm	45–48	32–35	16–18	12–15	0.7–1.55	150–500	248	3.2	25
Pineapple	70–82	-	5–12	14	1.5	105–300	170–1672	82	1–3
Sisal	67–78	10–14.2	8–11	10–22	1.45	50–200	468–700	9.4–22	3–7
Ramie	68.6–76.2	13.1–16.7	0.6–0.7	7.5–17	1.55	35–60	400–938	61.4–128	1.2–3.8
Rice	41–57	33	8–19	14	0.9–1.5	15–25	100–160	0.3–2.6	5.4–10.6
Wheat	39–45	15–31	13–20	18–20	1.1–1.3	20–40	90–150	0.2–2.2	3.5–6.6

**Table 3:** Comparison of WPC with commonly used materials.

Comparison of Materials	Climatic Conditions	Insulation	Lifespan	Installation	Environmental Protection
Bamboo	Poor	Poor	Vulnerable (1–2 Years)	Difficult	Somehow eco-friendly
Cement	Poor	Poor	Ease of loss	Very difficult	Causes pollution
WPC	Strong [47]	High	10 Years or more [48]	Easy installation	renewable and sustainable in nature [49]

## Data Availability

The data used in the study is available within the article.
